# Using selection models to assess sensitivity to publication bias: A tutorial and call for more routine use

**DOI:** 10.1002/cl2.1256

**Published:** 2022-07-01

**Authors:** Maximilian Maier, Tyler J. VanderWeele, Maya B. Mathur

**Affiliations:** ^1^ Department of Experimental Psychology University College London London UK; ^2^ Department of Psychology University of Amsterdam Amsterdam The Netherlands; ^3^ Department of Epidemiology Harvard University Cambridge Massachusetts USA; ^4^ Quantitative Sciences Unit, Department of Pediatrics Stanford University Stanford California USA

## Abstract

In meta‐analyses, it is critical to assess the extent to which publication bias might have compromised the results. Classical methods based on the funnel plot, including Egger's test and Trim‐and‐Fill, have become the de facto default methods to do so, with a large majority of recent meta‐analyses in top medical journals (85%) assessing for publication bias exclusively using these methods. However, these classical funnel plot methods have important limitations when used as the sole means of assessing publication bias: they essentially assume that the publication process favors large point estimates for small studies and does not affect the largest studies, and they can perform poorly when effects are heterogeneous. In light of these limitations, we recommend that meta‐analyses routinely apply other publication bias methods in addition to or instead of classical funnel plot methods. To this end, we describe how to use and interpret selection models. These methods make the often more realistic assumption that publication bias favors “statistically significant” results, and the methods also directly accommodate effect heterogeneity. Selection models have been established for decades in the statistics literature and are supported by user‐friendly software, yet remain rarely reported in many disciplines. We use a previously published meta‐analysis to demonstrate that selection models can yield insights that extend beyond those provided by funnel plot methods, suggesting the importance of establishing more comprehensive reporting practices for publication bias assessment.

## 1 INTRODUCTION

In meta‐analyses, publication bias—such as the preferential publication of papers supporting a given hypothesis rather than null or negative results—can lead to incorrect meta‐analytic estimates of the mean effect size. The funnel plot is among the most popular tools to assess evidence for the presence of publication bias and the sensitivity of results to publication bias (e.g., Duval & Tweedie, 2000; Egger et al., 1997). The funnel plot relates the meta‐analyzed studies' point estimates to a measure of their precision, such as sample size or standard error. Funnel plots are usually interpreted as indicating publication bias when they are asymmetric, that is, when smaller studies tend to have larger point estimates. Several popular statistical methods, such as Trim‐and‐Fill (e.g., Duval & Tweedie, 2000) and Egger's regression (Egger et al., 1997) are designed to quantify this type of asymmetry. When referring to “funnel plot methods,” we focus on these classical, very widespread approaches rather than on recent extensions that have not yet become common practice (Bartoš et al., 2021; Stanley & Doucouliagos, 2017; Stanley et al., 2017).

Among meta‐analyses in medicine that included some assessment of publication bias, nearly all do so using classical funnel plot methods. We reviewed meta‐analyses published in *Annals of Internal Medicine, Journal of the American Medical Association*, and *Lancet*. To do so, we systematically sampled 25 meta‐analyses by journal (75 total), reviewing the meta‐analyses reverse‐chronologically from 2019 back to 2017. (More details about the methodology and results of the review are available in the Supporting Information.) Among these meta‐analyses, 55% did not assess publication bias at all. Of the 45% that did assess publication bias, 85% did so using classical methods based on the funnel plot, such as visual inspection of funnel plots, Egger's regression, or Trim‐and‐Fill. The remaining 15% used methods such as the fail‐safe N (Rosenthal, 1986) or the test for excess significance (Ioannidis & Trikalinos, 2007). Unfortunately, none of these 75 meta‐analyses used selection models, an important and methodologically viable alternative that we discuss below. While our review focused on meta‐analyses in medical journals, there is evidence that reporting practices are similar in other disciplines (Ropovik et al., 2021).

## 2 THE NEED FOR OTHER METHODS: USES AND LIMITATIONS OF FUNNEL PLOT METHODS

Funnel plot methods can be useful to assess general “small‐study effects” (Sterne et al., 2011): that is, whether the effect sizes differ systematically between small and large studies. Such effects could arise not only from publication bias but also from genuine substantive differences between small and large studies (Egger et al., 1997; Lau et al., 2006). For example, in a meta‐analysis of intervention studies, if the most effective interventions are also the most expensive or difficult to implement, these highly effective interventions might be used primarily in the smallest studies. Funnel plot methods detect these types of small‐study effects as well as those arising from publication bias.

In practice, though, funnel plot methods are often used and interpreted specifically as means of assessing publication bias rather than as means of assessing these general small‐study effects. Thus, in this context of publication bias assessment, these methods have important limitations. First, they effectively assume that small studies with large positive point estimates are more likely to be published than small studies with small or negative point estimates. Second, typically, they effectively assume that the largest studies are published regardless of their point estimates (Rothstein et al., 2005, pp.75‐9‐9). This is because if publication bias does indeed operate in this manner, then in a meta‐analysis without publication bias, larger studies would cluster more closely around the true mean (i.e., the mean of all studies, whether published or unpublished) than smaller studies, but large and small studies alike would have point estimates centered around the true mean (Borenstein et al., 2011, p. 283). Thus, the point estimates would tend to form a symmetric “funnel” shape. In a meta‐analysis more severely affected by publication bias which favors a specific direction, the reasoning goes, small studies with small or negative point estimates would more frequently be omitted from the plot than small studies with large positive point estimates or large studies. This selective publication would lead to an asymmetric funnel shape in which the observed small studies tend to have larger point estimates than larger studies. As our review and work by others indicate (Ropovik et al., 2021), funnel plot methods essentially remain the sole means of assessing publication bias in a large majority of high‐profile meta‐analyses in different disciplines. However, echoing others' caveats (Lau et al., 2006; Sterne et al., 2011), we believe that this exclusive focus is problematic. As noted above, when funnel plot methods are interpreted as indications of and corrections for publication bias rather than as indications of general small‐study effects, the methods make implicit assumptions about how publication bias operates and about the distribution of effect sizes across studies. These assumptions are rarely stated in papers that apply funnel plot methods, and in many meta‐analyses, the assumptions may not align well with the way publication bias operates in practice.

Specifically, funnel plot methods assume that publication bias operates on the size of studies' point estimates regardless of their p‐values, yet empirical evidence suggests that selection for “statistically significant” p‐values (i.e., less than 0.05) is in fact a strong influence and likely stronger than selection for point estimate size (Masicampo & Lalande, 2012; McShane & Gal, 2017; Nelson et al., 1986; Rosenthal & Gaito, 1963, 1964; Wicherts, 2017). For better or for worse, in most fields, it is “significant” p‐values that researchers attempt to achieve and that attract the attention of peer reviewers and editors. Furthermore, in many meta‐analyses, it seems implausible that large studies are immune to publication pressures, a situation that further violates the assumptions of funnel plot methods. Funnel plot methods may fail to detect publication bias that operates on p‐values if such publication bias does not induce a strong correlation between studies' estimates and standard errors, or if publication bias selects for “significant” results in either direction.^1^ In either of these cases, publication bias might not induce asymmetry in the funnel plot. Besides their assumptions regarding the publication process, funnel plot methods can perform poorly when effects are heterogeneous across studies, as we detail below (e.g., Carter et al., 2019; Maier et al., 2022). For these reasons, funnel plot methods should be supplemented with selection models, or other comparable methods, so as to more effectively assess publication bias.

## 3 SELECTION MODELS AS AN ADDITIONAL MEANS TO ASSESS PUBLICATION BIAS

We are not opposed to using funnel plot methods for assessing general small‐study effects, but it is problematic to use them as the sole means of assessing publication bias, as is current practice in high‐profile medical meta‐analyses. Given the limitations of funnel plot methods, we believe that meta‐analyses should routinely apply other methods as well. As one reasonable alternative, selection models make more flexible assumptions regarding publication bias and heterogeneity that we believe are more realistic for the majority of meta‐analyses (Hedges, 1984; Iyengar & Greenhouse, 1988; Vevea & Hedges, 1995). For example, selection models can be specified to allow for publication bias that favors studies with “statistically significant” p‐values less than 0.05 and with point estimates in the desired direction, regardless of their sample sizes or the magnitude of their point estimates (Vevea & Hedges, 1995). Selection models of this form essentially assess whether, among the published studies, there is a relative overrepresentation of studies with significant p‐values and positive estimates compared to studies with nonsignificant p‐values or negative estimates. To do so, the models use maximum likelihood estimation to obtain a bias‐adjusted meta‐analytic mean by giving more weight to observed studies that are underrepresented in the sample due to publication bias (i.e., those with point estimates in the unexpected direction or with “nonsignificant” p‐values; Card, 2015, pp. 274–275). Additionally, selection models directly accommodate effect heterogeneity through the specification of, for example, a normal likelihood for the effect distribution (Rothstein et al., 2005; Terrin et al., 2003).

## 4 EXAMPLE—FUNNEL PLOT METHODS AND SELECTION MODELS CAN PROVIDE DIFFERENT INSIGHTS

**Figure 1:**
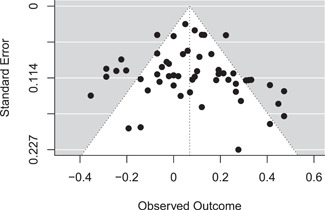
Funnel plot for Toosi et al. (2012). Correlations ("Observed Outcome”) are displayed after Fisher's Z transformation.

The differing assumptions of funnel plots versus selection models are not merely a point of statistical pedantry but rather can lead the methods to provide differing insights when applied to published meta‐analyses. For example, Toosi et al. (2012) meta‐analyzed studies on the effect of interracial interactions on positive attitudes, negative affect, nonverbal behavior and performance. A standard random‐effects model, before any adjustment for publication bias, indicates that performance was somewhat higher in dyads of the same race compared to dyads of different races, r=0.07 (95% confidence interval [CI]: [0.02, 0.12], p=0.003) with estimated heterogeneity $\hat{\tau }=0.15$ (95% CI: [0.12, 0.21]) and *I* ^2^ = 76% (95% CI: [66%, 87%]).^2^ The authors assessed publication bias using Egger's regression, finding little evidence for funnel plot asymmetry. In addition, visual inspection of the funnel plot did not seem to indicate publication bias (Figure 1).

We reanalyze the data with a simple selection model that assumes that “significant” studies, regardless of effect direction, are more likely to be published than “nonsignificant” studies. The R code to conduct this analysis this can be found in code excerpt (2), which we explain below. Our reanalysis suggests evidence for publication bias, $\chi^{2}\left(2\right)=11.82,p=0.003$. Accordingly, the estimated mean after adjusting for publication bias is smaller and has a larger p‐value, r=0.04 (95% CI: [−0.02,0.09], p=0.170). As noted above, these differing results could occur if, for example, there is little correlation between studies' estimates and standard errors (as detected by Egger's regression), yet there is an apparent overrepresentation of “significant” studies in either direction (as detected by the selection model). This example illustrates that using selection models to accommodate a more realistic mechanism of publication bias (i.e., selection for statistical significance that affects all studies, rather than selection for large point estimates that does not affect very large studies) and effect heterogeneity can result in different insights from those provided by funnel plot methods.

## 5 PRACTICAL RECOMMENDATIONS FOR APPLYING AND INTERPRETING SELECTION MODELS

Selection models are easy to implement in practice. The R package weightr includes a function to fit a selection model as follows (Coburn et al., 2019):

(1) $$\begin{array}{ccl} & & \mathrm{library(weightr)}\\ & & \mathrm{weightfunct(effect, v)}, \end{array}$$

where the argument “effect” represents the studies' point estimates and “v” represents their estimated variances (i.e., squared standard errors). By default, the weightr package assumes that studies with a two‐tailed p<0.05 and with positive estimates have a higher probability of publication than studies with p ≥ 0.05 or with negative estimates. Therefore, if the hypothesized direction of the effect is negative rather than positive, the signs of all point estimates should first be reversed before using this code. In this model of publication bias, effects in a particular direction are favored for publication. However, it is possible in some contexts that all “significant” results are favored regardless of direction; to fit a more flexible selection model that accommodates this possibility, the R code in code excerpt (1) could be modified as follows:

(2) weightfunct(effect, v, steps = c(0.025, 0.975, 1)).

The argument “steps” represents the p‐value cutoff points, representing the p‐values at which a result's probability of being published is thought to change. To facilitate specifying publication bias that selects for the estimate direction as well as the p‐value, the “steps” argument is specified in terms of one‐tailed, not two‐tailed, p‐values. We refer to one‐tailed p‐values as “$p_{+}$” to distinguish them from two‐tailed p‐values. That is, a one‐tailed $p_{+}<0.025$ represents a positive estimate that is “significant” on a *two*‐tailed test, whereas a one‐tailed $p_{+}>0.975$ represents a negative estimate that is “significant” on a two‐tailed test.^3^ The “steps” argument allows further customization of the selection model, such as allowing for selection based on one‐tailed rather than two‐tailed hypothesis tests or allowing for selection that also favors “marginally significant” results with a two‐tailed p<0.10 to a lesser degree than it favors “significant” results. However, in most scientific contexts, publication bias seems to conform well to one of the two specifications given in models (1) and (2) (Gelman & Carlin, 2014; Masicampo & Lalande, 2012; Mathur & VanderWeele, 2021; Wicherts, 2017).

In practice, we would recommend first fitting the more flexible model (2) to allow for the possibility of selection for both positive and negative “significant” results. If too few studies fall into one of the three categories (positive and “significant,” negative and “significant,” and “nonsignificant”), the function will issue a warning, in which case we would recommend then fitting the simpler model (1) after coding point estimates' signs such that the hypothesized effect direction is positive.

The function weightfunct reports the results of a standard random‐effects meta‐analysis without correction for publication bias, followed by analogous estimates after adjustment for publication bias. For example, for the meta‐analysis on interracial dyads, we used the R code in code excerpt (2) to fit a selection model that accommodates the possibility of selection for both positive and negative “significant” results. Based on this model, the estimated mean adjusted for publication bias is r=0.04 (95% CI: [−0.02,0.09], p=0.170). This p‐value corresponds to a test of the pooled estimate versus an effect size of 0 in the model adjusted for publication bias. The function also estimates “weights” for each interval of p‐values defined by the cutoffs. For the meta‐analysis on interracial dyads, for example, the estimated weight for the interval $0.025<p_{+}<0.975$ is 0.19. Because *one*‐tailed p‐values in this interval correspond to *two*‐tailed p‐values greater than 0.05 and with estimates in either direction, this estimated weight indicates that “nonsignificant” two‐tailed p‐values are estimated to be only 19% as likely to be published compared to “significant” two‐tailed p‐values with point estimates in the expected positive direction. For the interval $0.975<p_{+}<1$ (corresponding to two‐tailed p‐values less than 0.05 with negative estimates), the estimated weight is 1.05, indicating that studies that are “significant” but with point estimates in the unexpected negative direction are approximately as likely to be published as studies that are “significant” and positive. However, this estimate has considerable uncertainty because only six studies were “significant” and negative. Finally, the function provides a likelihood ratio test assessing for the presence of publication bias. We would suggest that meta‐analysts characterize the presence of publication bias in terms of the p‐value of this test and the severity of publication bias (assuming it is present) in terms of the estimated weights themselves, as we illustrate above. Other software packages are available for fitting similar selection models (e.g., Rufibach, 2015; Viechtbauer, 2010).

## 6 FURTHER EVIDENCE THAT FUNNEL PLOT METHODS ALONE ARE NOT ADEQUATE

Simulation studies suggest that funnel plot methods can perform poorly at detecting and correcting for publication bias. The methods may spuriously detect publication bias when in fact there is none (Type I error) or, inversely, fail to detect publication bias when it does exist (Type II error) (e.g., Carter et al., 2019; Pustejovsky & Rodgers, 2019). These findings regarding Type I error are corroborated by a recent analysis of registered replication reports (RRRs), a publication type in which an article receives “in principle acceptance” based only on the introduction and methods sections (Maier et al., 2022). In principle, then, RRRs should be subject to little (if any) selective reporting or publication bias (Chambers, 2013; Chambers et al., 2015). In an analysis of 28 RRRs, Egger's regression found evidence for publication bias in 9 of 28 data sets, again suggesting a high false‐positive rate. On the other hand, selection models did not find evidence for publication bias in any of these data sets (Maier et al., 2022).

These findings regarding inflated Type I and Type II error rates in part reflect the statistical assumptions of funnel plot methods, as discussed above. The inflated error rates can also occur when effects are heterogeneous across studies; in these settings, many funnel plot methods are prone to detecting publication bias even when none exists (Egger et al., 1997; Higgins et al., 2019; Lau et al., 2006; Pustejovsky & Rodgers, 2019). However, in practice, meta‐analyses often show moderate to high heterogeneity (Mathur & VanderWeele, 2021; McShane et al., 2016; Rhodes et al., 2015; van Erp et al., 2017), for example, because the effect size differs across participant populations (Rothstein et al., 2005). Additionally, the standard errors of many effect‐size measures (e.g., standardized mean differences) are arithmetically related to the effect size itself. This can induce an artifactual correlation between point estimates and their standard errors that funnel plot methods cannot distinguish from correlation induced by publication bias.^4^

## 7 DISCUSSION

Funnel plots and selection models can provide different insights in their assessments of publication bias, as we have illustrated using a previously published meta‐analysis. This largely reflects the methods' differing assumptions: funnel plot methods assume that publication bias operates based on effect sizes and standard errors and does not affect the largest studies, whereas selection models make the often more realistic assumption that publication bias operates on the “statistical significance” of p‐values. Furthermore, funnel plot methods do not directly accommodate effect heterogeneity, whereas selection models do.

Nevertheless, selection models are not a panacea, nor are they the only reasonable methods to supplement funnel plots when assessing publication bias. Like all statistical methods to assess publication bias, selection models do require statistical assumptions. Most selection model specifications assume that, before selection due to publication bias, the true effects are normally distributed, independent, and not correlated with the point estimates' standard errors, assumptions that are also standard in random‐effect meta‐analysis more generally. Publication bias may not always conform exactly to the assumed p‐value cutoffs, potentially compromising selection model estimates (Vevea & Hedges, 1995), but as we have discussed, empirical evidence suggests that the assumed model of publication bias often holds well in practice. Also, selection models perform best in large meta‐analyses and might fail to converge or provide imprecise estimates if there are few studies in one or more of the p‐value intervals (Carter et al., 2019; McShane et al., 2016; Terrin et al., 2003). In these settings, or when the normality or independence assumptions are considered implausible, it may be more informative to report sensitivity analyses that characterize how severe publication bias would hypothetically have to be to “explain away” the results of a meta‐analysis rather than attempting to estimate and correct for the actual severity of publication bias (Mathur & VanderWeele, 2020). In addition, Bayesian selection models can mitigate estimation problems in cases where few primary studies are available (Larose & Dey, 1998; Smith et al., 2001). There are also Bayesian publication bias adjustment methods that allow simultaneous application of recent extensions to funnel plot methods and selection models (Bartoš et al., 2021; Maier et al., 2022). While the discussion of these methods is beyond the scope of this manuscript, Bartoš et al. (2020) provided a tutorial paper with accompanying videos. Finally, some researchers have criticized selection models because published meta‐analyses adjusted via selection models can yield differing results from preregistered replication studies on the same topic (Kvarven et al., 2020). However, as we have noted (Lewis et al., 2022), these findings likely also reflect genuine differences in effect sizes between meta‐analyses and replication studies; as such, these findings do not necessarily imply that selection models perform poorly.

Instead of focusing exclusively on funnel plot methods, meta‐analysts should additionally (or alternatively) consider the results of selection models, as one reasonable alternative. These more comprehensive reporting practices would considerably improve our understanding of publication bias in meta‐analyses. We have provided concrete guidance on how to fit and interpret selection models using existing user‐friendly software, and we demonstrated that they can provide additional insights beyond those provided by funnel plot methods alone. In doing so, we hope to open the door to more widespread adoption.

## FUNDING

This study was supported by NIH grant R01 LM013866 and R01 CA222147; the NIH‐funded Biostatistics, Epidemiology and Research Design (BERD) Shared Resource of Stanford University's Clinical and Translational Education and Research (UL1TR003142); the Biostatistics Shared Resource (BSR) of the NIH‐funded Stanford Cancer Institute (P30CA124435); and the Quantitative Sciences Unit through the Stanford Diabetes Research Center (P30DK116074).

## REPRODUCIBILITY

All data and code required to reproduce the applied examples is publicly available: https://osf.io/37y9f/.

## Footnotes

1 Publication bias that favors “significant” results with positive estimates does induce some degree of association between estimates and standard errors, but this association is nonlinear (Stanley & Doucouliagos, 2014). Because funnel plot methods specifically detect *linear* correlations whereas selection models more precisely capture the nonlinear form of the association that arises under this type of publication bias that selects for statistical “significance,” selection models often have greater statistical power to detect publication bias (Pustejovsky & Rodgers, 2019). It is also possible for effect sizes to be arithmetically related to their standard errors, an issue we describe below.

2 For all analyses, we converted correlations to Fisher's Z scale and transformed them back to the correlation scale for reporting; however, the heterogeneity is reported on Fisher's Z scale directly. Some analysts might alternatively choose to meta‐analyze estimates directly on the correlation scale if the correlations are not close to 0 or 1, which can make the heterogeneity estimate more easily interpretable.

3 One can optionally pass one's own p‐values to weightfunct, but it is important to note that doing so will override the package's default calculation of p‐values that are consistent with the specified selection mechanism.

4 For effect‐size measures that are artifactually correlated with their standard errors, one can sometimes use a modified standard error calculation that reduces artifactual correlation (Harbord et al., 2006; Peters et al., 2006; Pustejovsky & Rodgers, 2019; Rücker et al., 2008). Doing so can improve the performance of funnel plot methods when there is some heterogeneity, but some such methods may still perform poorly under moderate or high heterogeneity (Harbord et al., 2006; Peters et al., 2006), and simulations indicate that selection models still tend to perform better overall (Pustejovsky & Rodgers, 2019).
